# Happiness, positive emotions, and subjective well-being in dementia

**DOI:** 10.3389/fneur.2024.1422354

**Published:** 2024-07-23

**Authors:** Carolin Miklitz, Klaus Fliessbach, Cornelia McCormick

**Affiliations:** ^1^Department for Old Age Psychiatry and Cognitive Disorders, University Hospital Bonn, Bonn, Germany; ^2^German Center for Neurodegenerative Diseases (DZNE) Bonn, Bonn, Germany

**Keywords:** ecological momentary assessment, experience sampling, living well with dementia, quality of life, episodic memory system, experiencing self

## Abstract

Research on non-cognitive features of dementia traditionally focusses on neuropsychiatric symptoms and challenging behavior and thus on negative aspects of the disease. Despite the clinical observation that many patients frequently report subjective well-being and often express positive emotions there is only little research on the definition, measurement and determinants of subjective well-being and happiness in people living with dementia. Furthermore, the few studies there are, examined happiness using retrospective questionnaires and the accounts of relatives or caregivers. However, in dementia, the experiencing self becomes more significant since past and future thinking are fading into the background. Here, we review the relative scarce literature in this field, discuss different psychological constructs and their applicability for dementia research, and suggest methods for measuring the addressed constructs in people with dementia. In particular, we propose methodology to study happiness and positive emotions in the experienced moment of the participants using ecological momentary assessments (EMA). We believe that adequate measures of momentary subjective well-being might become an important outcome parameter in clinical dementia trials beyond the currently used quality of life measures.

## 1 Introduction

Clinical dementia research traditionally focusses on cognitive deficits and associated neuropsychiatric symptoms. Although the presence and severity of the latter are strong determinants of caregiver burden, accelerated disease progression and negative impact of quality of life (1, 2) they might not fully explain the large variation in the expression of subjective well-being of patients. As clinicians, we observe a large variety of the overall emotional state of the patients with some displaying frequent anxiety and depression, but others regularly reporting positive emotions and happiness. Whereas positive emotions have been largely neglected, we believe that adequate measures of subjective well-being and happiness could serve as an additional dimension in the clinical categorization of dementia stages and might become important outcome parameters in clinical dementia care. Therefore, in this review, we will synthesize current endeavors to study subjective well-being in patients with dementia and try to outline a knowledge gap and novel ways to measure subjective well-being and happiness in patients with dementia.

One of our main assumptions is that neurodegenerative dementia, such as associated with Alzheimer’s Disease (AD), alters the ability for introspection and self-perception needed to evaluate overall life satisfaction due to the decline in core cognitive domains such as episodic memory. To explain, the episodic memory system supports memories which are specific in time and place (3). A prominent example are unique personal past events [episodic autobiographical memories (4–6)]. This memory system is neurally closely associated with the hippocampus and adjacent medial temporal lobe (MTL) structures (7), which are often impacted early in neurodegenerative dementias. Whereas traditionally, the MTL has been proposed to be exclusively supportive of episodic memory functions, we now know that the same neural system is crucial to support our ability to envision episodic future events (5), conjure up fictitious scenarios (8), or spend our time mind-wandering (9). The same is true for patients with dementia due to AD who have difficulties remembering specific, episodic autobiographical memories, envisioning scenarios set in the future or even conjure up fictitious mental events (10). With this in mind, it becomes more difficult for patients with MTL damage to integrate personal past events and imagined future events into one coherent life story (11). In fact, some say that patients with MTL damage “live in the permanent present tense” (12). In all, what becomes evident is that, the momentary, experiencing self in contrast to the remembering self (13) becomes more important when examining subjective well-being in people with MTL damage, such as neurodegenerative dementia. Thus, we assume that neuropathological changes associated with dementia and their reflected cognitive symptoms alter the way subjective well-being can be conceptualized and also will influence the ability to self-report relevant features of self-perception and quality of life (QoL).

Coming with this assumption in mind, we hope to offer the reader a different viewpoint on the historical perspective and contemporary definitions of the psychological constructs of happiness and subjective well-being as well as current efforts to examine these constructs in people with dementia. While an indepth description of the psychological constructs can be found elsewhere [see (14, 15) for very comprehensive reviews], we will focus this mini-review on a few excellent ongoing projects examining the determinants of QoL and living well with dementia. Therefore, we limit ourselves to the main contemporary endeavors and focus more on the experiencing, momentary assessment of subjective-wellbeing of people living with dementia. In that, we will discuss whether current measurements of happiness and subjective well-being can be applied in the same way to people with dementia and what may potentially be promising avenues for future research. Of note, this review is written with patients with AD in mind who suffer from a profound episodic memory loss. Nevertheless, our thoughts and proposals for future research can also be tested in other types of dementia.

## 2 Happiness in dementia

### 2.1 Can people with dementia experience happiness?

While in the introductory text of the world data base of happiness it has been proposed that “one cannot say whether a person is happy or not if that person is intellectually unable to construct an overall judgment” (16) there is all good reason, over and above clinical observations, to believe that people with moderate and even severe dementia can still experience positive emotions, happiness, and subjective well-being. Studies demonstrated that both the subjective emotional experience as well as the recognition of happiness are long preserved in AD (17, 18). In addition, successful experiments have employed autobiographical memory recall, film clips, photos, and music to elicit positive emotions and happiness (19, 20). Furthermore, turning to the neuroscience of positive emotions and happiness, Alexander et al. provide an overview of different cortical and subcortical structures associated with the experience of positive emotions, including the left prefrontal cortex, orbitofrontal cortex, anterior cingulate cortex and the insula as well as subcortical structures such as the nucleus accumbens, amygdala and ventral pallidum. These brain networks appear to be resilient toward neurodegenerative processes during healthy aging and do not overlap with the typical atrophy patterns seen in dementia-specific neurodegeneration (21). Taken together, there are many reasons to believe that examining positive emotions and happiness in dementia is a relevant, fruitful and viable effort.

### 2.2 A brief history on subjective well-being and happiness research in dementia

In the contemporary happiness research different terms have been used interchangeably to describe “the subjective appreciation of one’s life as a whole” [(22), please see Box 1 for the most prominent definitions]. Whereas generally, the definition “happiness” encompasses both intuitive affective appraisal and cognitively guided evaluation, some other approaches emphasize the immediate, experienced affect as the most important constituent of well-being. In view of progressive cognitive decline, as associated with dementia, this momentary experience of well-being gains particular importance.

BOX 1 **Definitions of relevant constructs.**

**Happiness:** Veenhoven defines happiness as evaluating one’s own life as favorable with the own appreciation relying on two sources: the affect and the thoughts. He discriminates between the *hedonic affective component* comprising the amount of pleasantness in feelings, emotions and moods, and *contentment*, the cognitive component, which describes the subjective perception of whether one’s own aspirations and wishes have been met in the past and present or will be met in future. The overall happiness (also termed *life satisfaction*) results from the synthesis of these two components (16).

**Objective Happiness:** Kahnemann distinguishes between “experienced well-being” and “evaluated well-being” and defines “objective happiness” as the temporal distribution of experienced affect (13). In this moment-based concept happiness is operationalized as the integration of the affective state of individuals at particular moments. In contrast to other concepts of well-being “objective happiness” does not include reports of global evaluations of the (recent) past and exclusively draws on immediate introspection. The term “objective” implies that objective rules are applied to evaluate the subjective experience of “objective happiness” (e.g., interpersonally comparable reports, reliable report of the valence of an experience) (23).

**Positive Emotions:** Positive emotions describe pleasant or desirable brief situational responses (including subjective experience, cognitive processing and physiological changes) evoked by either conscious or unconscious appraisal processes (24). As multicomponent responses positive emotions are distinct from pleasurable sensation and positive affect (which refers to consciously accessible, often more long-lasting feelings). Positive emotions are regarded as markers of overall well-being and happiness (25).

**Subjective well-being (SWB):** This concept was developed in 1984 by Diener and since then has been widely applied. Diener defines that “subjective well-being reflects an overall evaluation of the quality of a person’s life from his/her own perspective” (26) and describes four main components of SWB: *pleasant affect*, *unpleasant affect*, *life satisfaction* and *domain satisfaction* (27). SWB can be differentiated from other related, but distinct concepts. The central discriminative element in the definition of SWB is the *subjective* appreciation of one’s own life. Therefore, SWB—unlike other related concepts—does not include potential predictors of a good life (such as relationships, health).

**Quality of Life (QoL):** This multidimensional concept has been defined by the world health organization (WHO) as “individuals’ perceptions of their position in life […] in relation to their goals, expectations, standards and concerns” incorporating many different aspects including physical, emotional, social and material well-being as well as independency and personal beliefs (28). In literature a great variety of different QoL measures including dementia-specific QoL instruments can be found (29).

Besides happiness and SWB several different terms have been used interchangeably in empirical happiness research [e.g., affective well-being, emotional well-being (29)].

In dementia care, the early conceptual work of personhood in the 1990s by Kitwood and Bredin’s (30, 31) represents a milestone. In this concept, *personhood* is defined as “a standing or status that is bestowed upon one human being, by others, it implies recognition, respect and trust” and is independent from a person’s cognitive performance (31). For people with dementia, the preservation of personhood is regarded as the prerequisite for well-being (30). Kitwood and Bredin posit that relative well-being in dementia relies on the achievement of four subjective states: a sense of personal worth, a sense of agency resulting from (even the smallest) own choices, social confidence as the result of a welcoming social environment, and hope. Based on their own clinical experience, they propose 12 observable measures of behavior from which relative well-being can be derived [e.g., “the assertion of desire,” “initiation of social contact” and “humor” (30)]. Also in the early 1990s, Lawton began research on Quality of Life (QoL) in dementia and highlighted the importance of examining positive aspects of life in people with dementia instead of relying on deficit-based assessments of cognitive function, activities of daily life (i.e., ADL) and behavioral disturbances (32).

Building on the work of Kitwood and Lawton, numerous studies have investigated well-being in dementia with the majority of studies equating subjective well-being with QoL (33). One of the most prominent endeavors examining subjective well-being and QoL is the current British dementia strategy, entitled “Living well with dementia” which proposes a person-centered approach to address the needs of people living with dementia (34). Alluding to this strategy, in 2014 the “Improving the experience of Dementia and Enhancing Active Life” project (IDEAL), a longitudinal study exploring the impact of social and psychological factors on the possibility of “living well” with dementia, was launched. The first IDEAL study aimed to investigate the meaning of “living well” with dementia and factors associated with it using a mixed approach including qualitative (interviews during home visits) and extensive quantitative, questionnaire-based assessments (35). Building on this, the IDEAL-2 project intended to investigate trajectories of capability to “live well” during dementia progression and evaluated new measures to reflect the experiences of “living well” from a patient’s perspective (36). Important for our review here is the theoretical framework of how “living well” with dementia is defined in the IDEAL program, namely as the result of the interplay between “capitals, assets and resources, challenges and adaptation.” In this model, *capitals, assets and resources* encompass a person’s past experiences, abilities and current social, environmental, economic, physical and psychological (e.g., self-esteem, optimism) aspects. Further, *challenges* to “living well” with dementia are described as dementia-related symptoms, their impact on cognition, impaired functional ability and any other challenges faced (e.g., frailty, other health problems). Lastly, *adaptation* is understood as the interaction between resources and challenges and describes the ability to manage and cope with the challenges encountered. These different aspects are regarded as components which taken together generate an index to what degree a person “lives well” (35, 36).

### 2.3 Is “living well” with dementia equivalent to subjective well-being?

Thus, so far, the literature on positive psychology in the field of dementia research has focused on personhood, QoL, and “living well.” While no unique operational definition of “living well” stands out there appears to be an overall agreement that “living well” in dementia cannot entirely be reflected by QoL measures alone (37). Different reviews aimed to identify key themes underlying well-being in dementia and consistently determined a preserved selfhood, a sense of agency, social connectedness and the experience of positive emotions as important aspects (37, 38). Some authors propose that the level to which a person with dementia “lives well” is reflected by a combination of subjective evaluations of QoL, life satisfaction and well-being (35, 37). Following this notion of “living well” which resembles Seligman’s interpretation of well-being as a construct with several measurable elements each contributing to it but none defining it (39) subjective well-being (SWB) could be regarded as one component of the broader concept of “living well” with dementia. Despite putting particular emphasis on the subjective evaluation of different components of “living well,” this concept still includes measures which could be regarded as objective predictors of well-being (e.g., QoL) rather than representing an individual expression of well-being itself. Moreover, similar to concepts of well-being from the general population, measures of QoL and life satisfaction which have been proposed as indicators of “living well with dementia” (35) not only require intuitive affective appraisal but also cognitively guided evaluation, partly relying on the integration of personal past and imagined future events.

Together, there are excellent and thoughtful projects to investigate which factors might contribute to “living well” with dementia. However, the main focus of our thoughts is not so much the life situation of a person with dementia and how this influences overall life satisfaction. Our main interest lays at the momentary experience of people with dementia and ways to assess these fleeting moments of happiness and well-being, regardless of the overall personal life situation. From the broader concept of “living well” with dementia, we are interested to step back and propose to closely examine the subjective well-being of individual moments.

### 2.4 The importance of the momentary assessment to measure happiness in dementia

As set out in the introduction, people with cognitive decline may have marked deficits in remembering their past or envision their future in a detailed, concise manner which may also impact their self-identity. Therefore, to assess happiness and subjective well-being in dementia, we believe that the assessment of momentary well-being is key. Prior research on the “lived experience in dementia” identified three key themes associated with “living well” with dementia: The experience of positive emotions in the immediate present, personal strengths and strategies (including humor and hope) to cope with the disease, and personal beliefs (38). In the book “Positive psychology approaches to dementia” Phinney points out that factors one might expect to be important in the consideration of “living well” could be experienced differently by people living with dementia. Reviewing qualitative research exploring the subjective experience of people with dementia she derives a notion of “living well” which includes three important aspects: First, preserved self-hood and identity as a result of inclusion and respect, second, socially significant purpose and activities resulting in a sense of belonging and third, pleasure (the experience of positive emotions) that often grows from meaningful engagement building on the “freedom to choose.” Based on this review, well-being is conceptualized in terms of bidirectional social relationships, interactions and emotional attachment. Furthermore, Phinney notes that moment-to-moment experiences of well-being might be the most important aspect of well-being for people living with dementia (40).

### 2.5 The promising tool of experience sampling techniques and ecological momentary assessment

In this context, research methods tapping into the momentary assessment of experiences become relevant. One of the most prominent techniques are ecological momentary assessments (EMA) and experience sampling [these terms are often used interchangeably (41)]. EMA refers to a method of data collection in which participants respond to a repeated assessment at specific moments over the course of time while functioning within their natural settings (42). Nowadays, in a typical EMA experiment, participants receive a random reminder via their mobile phone (e.g., 6×/day for 6 weeks) and are prompted to answer some quick questions about their mood, thoughts or actions. For example, a previous EMA study examined happiness in the youth by prompting students 8×/d for 1 week to answer open-ended questions about what they were doing as well as multiple-choice questions regarding whom they were with and close-ended scales addressing a wide range of feelings and conditions associated with that moment. The authors found that feeling good about one self, being excited, proud, sociable, and active are the strongest predictors of trait happiness (43). We propose to use EMA directly in patients suffering from neurodegenerative dementia to assess their version of momentary subjective well-being. In its simplicity, EMA overcomes some crucial hindrances in examining happiness in dementia research (44–46). In fact, since only the momentary mood is assessed, there is no need to integrate various lifelines or to think about ones’ assets, financial state or other life conditions. Having pointed out the various advantages of EMA tools in the assessment of momentary subjective well-being in dementia, several limitations have to be addressed. First, frequent assessments might increase patient burden. Second, persons with advanced cognitive impairment might have difficulties in the use of digital tools. Third, EMA relies heavily on introspective abilities which can be distorted in patients with dementia.

In literature, experience sampling has been successfully implemented to track depressive symptoms in people with moderate to severe dementia (44). In one experiment, 12 participants with advanced dementia were enrolled from an inpatient psychogeriatric unit. Over the course of 6 weeks, research staff observed and noted the mood of patients up to four times a day, 7 days a week. In addition, patients rated their own mood (i.e., sadness and anxiety) with a yes or no response. The authors found that patients were well able to express their mood consistently and in accordance to the observations made by the research staff (thus tapping into the “objective emotion” definition of Kahnemann, Box 1). Furthermore, another relevant EMA study examined aspects of daily life which are related to QoL in dementia (45). In addition to social and physical activity, better mood ratings turned out to be a strong predictor of higher QoL levels.

These studies demonstrate two important points. First, EMA is feasible in patients with severe cognitive impairment. Second, reference to observable, objective measures of momentary well-being assessed by proxies or research staff may add valuable information to understand to what extent the subjective well-being rated by dementia patients themselves is consistent with their well-being evaluated by observers.

Together, while there are no EMA data up to date on happiness in dementia, we believe that EMA is a promising future avenue to assess happiness, positive emotions, and subjective well-being in dementia across different dementia severity levels.

### 2.6 Avenues for future research toward exploring happiness in dementia

As set out in the introduction, as clinicians, we observe that patients with dementia often report positive emotions and subjective well-being. In the general population several lines of evidence indicate that high subjective well-being causes better health and longevity (46). However, it is unknown if this also holds true in dementia. In view of progressive cognitive deficits, we regard momentary experiences (including the experience of agency, social confidence and pleasure) as the most significant constituents of subjective well-being in dementia. We therefore believe that a systematic assessment of the momentary subjective well-being will gain a deeper understanding of the variety of emotions associated with dementia and its clinical implications. We propose evaluation methods which could be used as indicators of momentary subjective well-being in dementia (please see Table 1 for selected measures) and believe that because of the profound cognitive deficits related to the neuropathological changes associated with AD, EMA tools may be a promising new avenue for the most pressing questions going forward.

**Table 1:** Measuring indicators of well-being.

Measures	Categories assessed	Mode	No of items	Time frame
Satisfaction with life scale	Life close to one’s ideal	SR	5	Presence
[Diener et al., 1985 (47)]	Life conditions			
	Life satisfaction			
	Availability of “important things” in life			
	Changes in life if one could live life over			
Scale of positive and negative Experience (SPANE)[Diener et Biswas-Diener, 2009 (48)]	Positivity/negativity	SR	12	Past 4 weeks
	Feeling good/bad			
	Pleasantness/unpleasantness			
	Happiness/sadness			
	Joyfulness/fear			
	Contentment/anger			
World Health Organization-Five Well-Being Index[WHO, 1998 (49)]:	Cheerfulness	SR	5	Last 2 weeks
	Calmness and relaxation			
	Feeling active			
	Feeling of waking up recreated			
	Evaluation of life as interesting			
My life questionnaire	Agency	SR	10	Presence
[Clare et al., 2023 (50)]	Interpersonal relationships			
	Engagement			
	Activity			
	Sleep			
	Living situation			
	Social confidence			
**“**Life is meaningful to me Scale”	Happiness	SR	4	Day before
[Measures of personal well-being, ONS, 2018 (51)]	Anxiety			
	Worthwileness			Presence
	Overall life satisfaction			
12 observable measures of relative well-being[Kitwood et Bredin, 1992 (30)]	Pleasure	PR	12	Presence
	Relaxation			
	Agency			
	Social confidence			
	Worthwileness			
	Humor			

SR, self-rated; PR, proxy-rated. A detailed overview of observational and self-report measures of well-being in dementia studies can be found in Tinkler and Hicks, Clarke at al., and Madsø (51–53).

These are:

- Can we use EMA to assess and quantify the moments during which people with dementia report happiness, positive emotions, and subjective well-being? Can these moments be assessed across different stages of the AD continuum, such as Mild Cognitive Impairment (MCI), mild, moderate and severe stages?
- Which neural networks are associated with momentary subjective well-being and how are these networks affected by neurodegenerative diseases, such as AD?
- How are self-reported, momentary experiences of positive emotional states related to other indicators of subjective well-being such as agency, social confidence and worthwhileness?
- In the long run, can we establish parameters with which to track happiness in dementia? Are these parameters associated with disease progression and might they serve as outcome parameters in clinical trials?

## 3 Conclusion

Due to the profound cognitive deficits in remembering detail-rich past events and envision episodic future scenarios, patients with dementia due to AD have limited access to their individual evaluation of their life satisfaction. Therefore, we argue that the experienced moment of daily living should have the center stage in evaluating a persons’ self-expressed happiness and subjective well-being. Several methods, such as ecological momentary assessment have been successfully used in moderate to severe dementia. Thus, we outline future research avenues to assess happiness, positive emotions, and subjective well-being in patients across AD stages and believe that moment-based measures of subjective well-being might become an important outcome parameter in clinical dementia trials beyond the currently used quality of life assessments.

## Author contributions

CMi: Conceptualization, Visualization, Writing – original draft, Writing – review & editing. KF: Conceptualization, Supervision, Writing – original draft, Writing – review & editing. CMc: Conceptualization, Supervision, Writing – original draft, Writing – review & editing.
